# Communicating Science: Press Releases at *EHP*

**DOI:** 10.1289/ehp.1001913

**Published:** 2010-02

**Authors:** Jane C. Schroeder

**Affiliations:** *EHP*, E-mail: schroederjc@niehs.nih.gov

As a service to our contributors and the public, *EHP* issues occasional press releases to publicize papers that we believe are relevant and important to researchers, educators, public health practitioners, policy makers, and/or the general public, as well as to regular *EHP* readers and contributors. Specifically, our goal is for *EHP* press releases to serve science and the public by increasing awareness of work that might otherwise be overlooked by people who would benefit from reading it. Of course, press releases may also benefit authors and journals by increasing recognition and citation, which may in turn increase authors’ ability to obtain funding and advance their careers, and increase the journal’s readership and ability to attract high-quality submissions (Altman 1995; Barbour et al. 2008; Ransohoff and Ransohoff 2001; Shuchman and Wilkes 1997; Smith 2006; Stryker 2002). However, neither the lofty nor the practical benefits of press releases are realized unless they stimulate media coverage, and this requirement creates an incentive to produce attention-getting press releases that may fail to provide a balanced and realistic presentation of the implications of the research. Such press releases contribute to poor-quality health reporting and, in some cases, may do more harm than good (Altman 1995; Barbour et al. 2008; Bubela and Caulfield 2004; Ransohoff and Ransohoff 2001; Stryker 2002). With this in mind, we feel it is important to periodically evaluate *EHP*’s standards for press releases; we would like to take this opportunity to clarify our standards for our readers and contributors.

“Exaggeration serves many interests, but it does not serve the public’s interest. And in the end, it is self-defeating, because it undermines the credibility of medical science. After a while people may not believe anything we have to say.”

Schwartz and Woloshin 2003

Press releases provide an important link between scientists and journalists. Results of a 2005 survey of 468 health and medical science journalists indicated that press releases or press conferences were the initial source of ideas for 40–50% of news stories (Viswanath et al. 2008), and a review of 500 health news stories reported that 45% did not “go beyond a news release,” indicating that press releases were the only source of information in many cases (Schwitzer 2008). Research papers associated with press releases receive more media coverage and citations than other papers, and at least part of the difference in citations appears to be driven by the media coverage itself, independent of other characteristics of the publicized research (Phillips et al. 1991; Stryker 2002). Journal editors therefore influence the nature and scope of media coverage by choosing which papers to publicize (Stryker 2002; Viswanath et al. 2008). These choices may not only influence public perceptions but may also influence the actions of regulators and legislators (Smith 2006).

The impact of press releases on media coverage and science communication highlights the need to ensure that releases are accurate, complete, and clear, but systematic reviews of press releases issued by academic centers (Woloshin et al. 2009) and medical journals (Woloshin and Schwartz 2002) have demonstrated a tendency for press releases to exaggerate the importance of findings while failing to discuss study limitations or conflicts of interest. Consequently, it is not surprising that many news articles concerning health research also receive unsatisfactory ratings when evaluated using similar criteria (Schwitzer 2008). Journalists have developed standards to maintain or improve the quality of their reporting, as exemplified by the Association of Health Care Journalists’ Statement of Principles, which urges medical and science news writers to avoid vague and sensational language, acknowledge uncertainty, and clearly distinguish between results that represent associations versus causal relations (Schwitzer 2004). Scientists and journals also share responsibility for the quality of science reporting and can do their part to help ensure it by providing journalists with accurate and appropriate information in press releases and interviews (Ransohoff and Ransohoff 2001; Schwartz and Woloshin 2004; Shuchman and Wilkes 1997; Woloshin and Schwartz 2002).

Communicating the findings of environmental health research is central to *EHP*’s mission. Science communication to a broad audience may be facilitated by translation and reframing, but we also have a responsibility to communicate in a way that will help improve the public’s ability to understand the implications of environmental health research. With this in mind we will continue to strive to write press releases that present *EHP* papers in a meaningful and accurate way by putting findings into context without inappropriate extrapolation or exaggeration, and by providing key information on current knowledge, research methods, study limitations, and potential conflicts of interest (Woloshin and Schwartz 2002; Woloshin et al. 2009). This approach may not produce the most sensational headlines, but we believe it will increase the quality and long-term benefits of the media coverage that papers published in *EHP* receive.

## Figures and Tables

**Figure:**
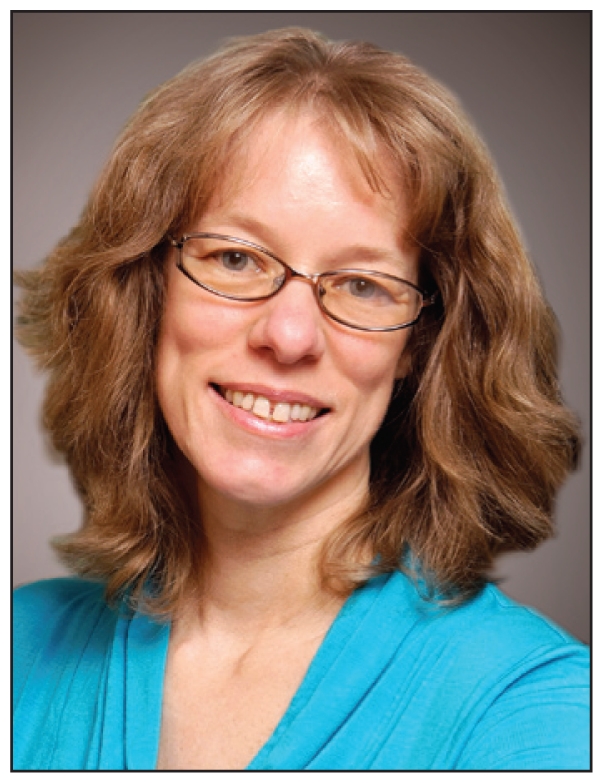
Jane C. Schroeder
